# SMOG 2: A Versatile Software Package for Generating Structure-Based Models

**DOI:** 10.1371/journal.pcbi.1004794

**Published:** 2016-03-10

**Authors:** Jeffrey K. Noel, Mariana Levi, Mohit Raghunathan, Heiko Lammert, Ryan L. Hayes, José N. Onuchic, Paul C. Whitford

**Affiliations:** 1 Center for Theoretical Biological Physics, Rice University, Houston, Texas, United States of America; 2 Kristallografie, Max Delbrück Center for Molecular Medicine, Berlin, Germany; 3 Department of Physics, Northeastern University, Boston, Massachusetts, United States of America; UCSD, UNITED STATES

## Abstract

Molecular dynamics simulations with coarse-grained or simplified Hamiltonians have proven to be an effective means of capturing the functionally important long-time and large-length scale motions of proteins and RNAs. Originally developed in the context of protein folding, structure-based models (SBMs) have since been extended to probe a diverse range of biomolecular processes, spanning from protein and RNA folding to functional transitions in molecular machines. The hallmark feature of a structure-based model is that part, or all, of the potential energy function is defined by a known structure. Within this general class of models, there exist many possible variations in resolution and energetic composition. SMOG 2 is a downloadable software package that reads user-designated structural information and user-defined energy definitions, in order to produce the files necessary to use SBMs with high performance molecular dynamics packages: GROMACS and NAMD. SMOG 2 is bundled with XML-formatted template files that define commonly used SBMs, and it can process template files that are altered according to the needs of each user. This computational infrastructure also allows for experimental or bioinformatics-derived restraints or novel structural features to be included, e.g. novel ligands, prosthetic groups and post-translational/transcriptional modifications. The code and user guide can be downloaded at http://smog-server.org/smog2.

This is a *PLOS Computational Biology* Software Article.

## Introduction

The study of biomolecular folding has produced theoretical concepts that are generalizable to many processes, such as conformational rearrangements in proteins and the functional dynamics of molecular assemblies. In particular, the principle of minimal frustration [1] and the folding funnel concept [2, 3] describe an energy landscape where the native interactions (i.e. the molecular interactions present in low free-energy configurations of folded proteins and RNAs) are on average more stabilizing than non-native interactions. Thus, the effective energetics of a biomolecule can be well described by a set of stabilizing native interactions, along with excluded volume to prevent chain crossing. Potential energy functions of this type are known as “structure-based models,” (SBMs) and they are powerful tools for probing the relationship between structure, folding and function in biomolecular systems. The simplified character of the potential energy function allows for reduced computational requirements, and the explicitly-encoded native interactions provide a baseline model for molecular modeling, or for studying physical perturbations. For a detailed discussion of the theoretical foundation and applications of SBMs, the reader is referred to the following reviews [4, 5] and the references therein.

SBMs were first extensively used to explore the predictions of energy landscape theory in the context of protein folding [6–15]. These studies showed that minimally-frustrated protein models reproduce many thermodynamic features of real proteins, and the predicted transition state ensembles are frequently consistent with experimental findings [8, 9, 16–18]. In addition to folding, studies used SBMs to show that protein binding could be understood within a common theoretical framework [19, 20]. Since protein function is governed by the same energy landscape that determines folding dynamics [21], these models have also been used to study the conformational dynamics involved in macromolecular function, e.g. adenylate kinase [22], kinesin [23, 24], and the ribosome [25]. These models have structural resolutions that vary from a single bead per residue [10], to all heavy atoms being explicitly represented [26], and their energetic complexity varies from “perfectly-funneled” landscapes, to Hamiltonians that include various flavors of non-native interactions [27–29]. Recently, SBMs have found utility in molecular modeling applications. For example, MDfit combines SBMs and cryogenic electron microscopy data to create atomically-grained structural models that are consistent with experimental electron densities [30]. Another example is SBM+DCA, where SBMs include co-evolutionary residue-residue interactions to predict difficult-to-crystallize oligomers [31, 32]. Together, SBMs (sometimes called “Go-models” [33]) have a thirty year history that spans countless applications, where the common feature is that biomolecular contacts present in high-resolution structures are given stabilizing energetics.

With the versatility of SBMs, investigators often apply customizations that are tailored to address specific physical questions. This contrasts with the more linear development of empirical explicit-solvent potentials, which is driven by the reproduction of experimental observables for model systems. As a result, SBM development has been decentralized, which has limited the portability and transferability of the models. Web servers [34, 35] that produce output for running SBMs on modern molecular dynamics (MD) packages have been very popular, and have provided some degree of standardization. However, since these web servers only provide the specific variations of the models that the developers decide to support, modifications made by the general community are typically unavailable to other researchers.

SMOG 2 is intended to facilitate SBM development by allowing modifications and extensions to be easily shared by the research community. In SMOG 2, an SBM potential is translated into a template format, allowing forcefields to be easily disseminated and modified. SMOG 2 processes user-designated structural information provided in standard Protein Data Bank (PDB) format and a SMOG 2 template, in order to generate the forcefield files required to perform simulations with MD platforms. Two of the most widely used MD platforms, GROMACS [36] and NAMD [37], support SMOG 2 output files. SMOG 2 is licensed under the GNU GPL and the source code is publicly available. See http://smog-server.org/smog2 for details and the user guide.

## Design and Implementation

### Template-based design

Many functional biological macromolecules are polymers of amino acids or nucleic acids, the building blocks of proteins, RNA and DNA. Each residue has a unique set of atoms, called the side chain in proteins (or base in nucleic acids), and a common set of atoms that constitutes the polymer backbone. Thus, an intuitive approach for defining the covalent connectivity within a biomolecule is to predefine the covalent structure of each possible residue and then map these interactions on a per-residue basis. Conditions must also be provided that ensure adjacent residues are covalently linked. In addition, generic non-bonded (non-covalent) interactions between atoms can be defined by assigning each atom a chemical “type” and then specifying the functional forms of the interactions between all possible combinations of types. This approach is sufficient to describe any polymer sequence composed of the predefined building blocks. SMOG 2 adopts this strategy for defining SBMs, which is consistent with the organization used for semi-empirical models, such as AMBER [38], CHARMM [39], and GROMOS [40]. This consistency in the construction of the models also allows the interactions defined in semi-empirical models to be mapped to SMOG 2 syntax in order to construct hybrid-variants of these models.

SMOG 2 templates are written in XML (eXtensible Markup Language) for readability and standardization. A SMOG 2 template, which defines a specific forcefield, is comprised of four files with the following suffixes:

- **.bif**: Defines the atoms and bonds in each residue and their connectivity. Any atom names may be used, though the naming between the .bif and input PDB file must be consistent.
- **.sif**: Defines the available functional forms for interaction potentials and system-wide energetic settings.
- **.b**: Sets the specific functional forms to be applied for bonded interactions between atom types.
- **.nb**: Sets the specific functional forms to be applied for non-bonded interactions between atom types.

The included templates (see section **Included templates**) follow standard PDB nomenclature for simplicity. Internally, the code makes no assumptions about the molecular structure corresponding to specific residue names or the interactions associated with specific atom names. Thus, adding new ligands and residue types involves defining the consituent atom names and their covalent bonds in the .bif. Each atom has three associated “types” that can be used to control the interactions between atoms: bonded-type, non-bonded-type, and pair-type. These parameters define how to map the bonded interactions, non-native non-bonded interactions, and native contact interactions, respectively. It should be pointed out that irregular molecular chains (i.e. without a common backbone) such as polysaccharides cannot be automatically handled. To accommodate for these types of irregular chains, the inter-residue bonds must be explicitly defined in the PDB file, as described in the SMOG 2 manual.

#### SBM Hamiltonians are defined by the input structure

The main purpose of SMOG 2 is to facilitate the creation of input files for simulations that contain structure-based interactions. For our purposes, a “structure-based interaction” is an interaction that is parameterized by the atomic coordinates of a known, low-free-energy configuration (e.g., an X-ray crystallographic structure). In a “pure” SBM, the global minimum of the Hamiltonian is encoded as the configuration of the input (native) structure by explicitly defining the native value of each interaction to be the potential energy minimum. For illustration, consider the Hamiltonian of a commonly used coarse-grained SBM [7], where each protein residue is represented by a bead at the position of the C_*α*_ atom:

$$\begin{array}{lll} H_{\text{C}\alpha }\left(\vec{x},{\vec{x}}^{0}\right) & = & \sum_{ij\in \text{bonds}}\frac{\epsilon_{\text{b}}}{2}{\left(r_{ij}-r_{ij}^{0}\right)}^{2}+\sum_{ijk\in \text{angles}}\frac{\epsilon_{\theta }}{2}{\left(\theta_{ijk}-\theta_{ijk}^{0}\right)}^{2}+\sum_{ijkl\in \text{dihedrals}}\epsilon_{\text{D}}F_{\text{D}}\left(\varphi_{ijkl}-\varphi_{ijkl}^{0}\right)\\ & + & \sum_{ij\in \text{contacts}}\epsilon_{\text{C}}\left[5{\left(\frac{r_{ij}^{0}}{r_{ij}}\right)}^{12}-6{\left(\frac{r_{ij}^{0}}{r_{ij}}\right)}^{10}\right]+\sum_{ij\notin \text{contacts}}\epsilon_{\text{NC}}{\left(\frac{\sigma_{\text{NC}}}{r_{ij}}\right)}^{12}. \end{array}$$ (1)

The dihedral potential *F*_D_ is

$$F_{D}\left(\delta \varphi \right)=\left[1-\cos\left(\delta \varphi \right)\right]+\frac{1}{2}\left[1-\cos\left(3\delta \varphi \right)\right].$$

The backbone structure is maintained by harmonic bonds and angles, the secondary and tertiary structure is stabilized by dihedral and short-range contact potentials, and all beads interact through an excluded volume interaction. Contacts are defined as being between residue pairs that are in spatial proximity in the native structure [41]. The superscript 0 denotes that a parameter is calculated from the input structure, which is used to explicitly set the global minimum of the potential to the input configuration.

#### Including native structural information and coarse-graining

SMOG 2 was written specifically for Hamiltonians of the general form shown in Eq 1. In the SMOG 2 templates a question mark is used to indicate that a parameter should be calculated from the native structural information. For example, in the .b file for the Hamiltonian in Eq 1, the bond function would be declared as:

<bond func=“bond_harmonic(?,20000)”>

<bType>*</bType>

<bType>*</bType>

</bond>

This specifies that a harmonic bond potential with *ϵ*_b_ = 20000 be given between an atom pair *ij* of the indicated bonded types (bType). In this case, the asterisks stipulate that this function be applied to all bType combinations that are not explicitly defined elsewhere in the .b file. Note that the units should be consistent with how GROMACS implements reduced units; for a more detailed discussion, see the user manual. As noted above, a unique feature of SMOG 2 is the question mark special character. In this example, the question mark specifies that the native distance $r_{ij}^{0}$ should be used to define the minimum of the harmonic potential. This question mark syntax can be similarly used for any interaction term in Eq 1. To provide a parameter that is independent of structure, such as *σ*_NC_, which defines the excluded volume between the beads, a numerical value should be provided in place of a question mark.

SMOG 2 implements automatic coarse-graining by using two templates internally, one atomistic template that is consistent with the input PDB structure, and one coarse-grained template. The coarse-grained template specifies one atom within each residue to map interactions and include in the simulation model. This feature is useful for creating single-bead models of proteins, such as the commonly-used C_*α*_-model of Clementi, Nymeyer and Onuchic [7]. Coarse-grained geometries differing from a single-bead-per-residue representation can be implemented by creating a template consistent with a preprocessed PDB structure containing only the coarse-grained atoms. Note that the Shadow.jar contact map generation tool is only intended for use with a structure containing all the heavy atoms. Thus, for general coarse-graining, the native contacts will have to be mapped onto the coarse-grained atoms by the user and be provided to SMOG 2 as input.

#### Included templates

SMOG 2 is packaged with templates for some commonly used structure-based Hamiltonians [7, 26, 42–46] (Table 1). These templates can be used as-is or modified to create new SBM variants. Users that generate new templates are encouraged to make them publicly available through the SMOG webpage. This can help provide transparency and encourage collaboration.

**Table 1:** Description of the SMOG 2 templates included in the distribution. Except where noted, the native contact map is generated by the Shadow algorithm [41] using an input all-atom PDB structure. The elastic network model is in the same spirit as Tirion’s [46], but the contact map is different and the spring stiffness is system independent.

Template	Ref.	Description
SBM_AA	[26]	All heavy atoms explicitly represented, Lennard-Jones potentials for native atomic contacts, handles RNA/DNA/protein/ligands
SBM_AA+gaussian	[42, 43]	SBM_AA with Gaussian potentials for native atomic contacts
SBM_AA_charged	[44]	SBM_AA with charged ARG, LYS, GLU, ASP, N/C-terminal
SBM_CA	[7]	Single C_*α*_ bead per residue, Lennard-Jones potentials for native residue contacts, developed for proteins
SBM_CA+gaussian	[45]	SBM_CA with Gaussian potentials for native residue contacts
ENM		All-atom elastic network model, harmonic potentials for native atomic contacts, 6 Å cutoff determines native contact map

### Code implementation

SMOG 2 is written in the Perl programming language, and it uses the Perl Data Language (PDL) for its primary data structures. PDL extends the native Perl data structures by allowing for large multidimensional arrays that can be manipulated through vector-based operations. PDL arrays are more compact, and can be manipulated faster than native Perl arrays. This is important for the most computationally intensive task performed by SMOG 2, which is to dynamically calculate all angles and dihedrals that can exist in a molecule based on the bonded geometry. The Perl implementation has a few dependencies: String::Util, XML::Simple, Exporter, and XML::Validator::Schema. Additionally, the Java Runtime Environment (JRE 1.7 or greater) is necessary for SMOG 2 to call the included Shadow.jar contact map tool [41].

In order to ensure that SMOG 2 is properly configured, test modules are available to the user as a separate download (smog-check) from the SMOG website. The smog-check bundle contains two test programs. One is a basic check that ensures that the local installation reproduces benchmark output files (.top, .gro). The second testing suite is a rigorous test-driven-development package that inspects the output of SMOG 2 for accuracy after code modifications. SMOG 2 has been extensively beta tested and exception-driven-development (i.e. checking for previously encountered errors and providing feedback to the user on how to correct the errors) has been implemented throughout the code.

### Workflow

SMOG 2 is invoked from the command line. The two necessary inputs are 1) a biomolecular structure in PDB format and 2) a directory name containing the set of SMOG 2 templates. Users are encouraged to use the included tool smog_adjustPDB, which resolves common formatting/naming inconsistencies between standard PDB format and the default templates. The templates define the general form and parameters of the Hamiltonian. SMOG 2 can process default templates that are included within the package (Table 1), as well as user-generated templates. The native contact map can be either automatically generated or provided as input. Running SMOG 2 generates output files that are formatted for input to MD software packages (in GROMACS format, which can also be read by NAMD). At a minimum, two of the generated files are required in order to run a simulation:

- **.gro**: The coordinates of the input PDB structure in GRO format. This is often used as the initial configuration for MD simulations.
- **.top**: The topology file specifies the Hamiltonian by listing all atomic interactions.

The user’s manual and the SMOG web server [34] both provide tutorials for using the generated files to perform MD simulations. While SMOG 2 can be used to generate a wide range of possible models, for some extended SBM variants, it will be necessary to further process the topology files. For example, combining multiple SBMs into a multi-basin landscape is a commonly used technique that is not automatically handled by SMOG 2. This task and other useful post-processing of topology files can be performed with the Python-based eSBMTools [47].

## Results and Discussion

### Protein folding with the default models

**Fig 1:**
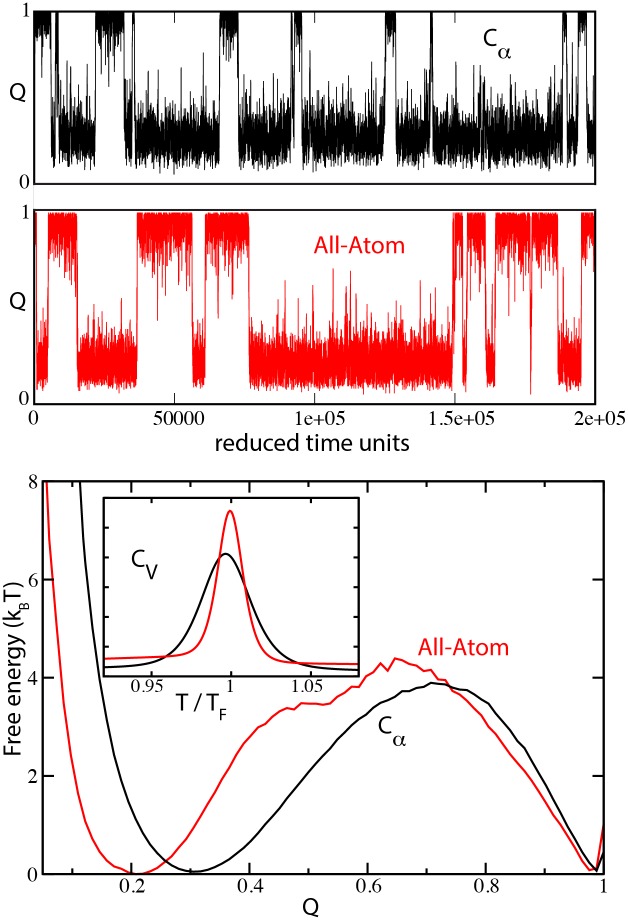
Protein folding simulations with the default C_*α*_ and all-atom models of the 64 residue chymotrypsin inhibitor 2 (PDB code: 1YPA). Top: Folding trajectories near folding temperature (T_F_) of the C_*α*_ (black) and all-atom (red) models. Bottom: Free energy as a function of $Q_{C\alpha }$, the number of native C_*α*_ pairs within 1.5 times their native distance. The same coordinate is used to describe both models. Inset: Specific heat for the two models (normalized to have equal area). T_F_ in reduced units for the all-atom model is 0.97 and for the C_*α*_ model is 1.17 (117 and 140 in the GROMACS .mdp file, respectively).

As an illustration of the types of SBM variants that can be explored with SMOG 2, folding simulations of the well studied protein chymotrypsin inhibitor 2 (CI2) [48] are considered. The results for two different models are shown, a single-bead-per-residue graining [7] and an all-heavy-atom graining [26] (SBM_CA and SBM_AA in Table 1, respectively), using the input run parameters suggested in the user’s manual (Fig 1). A standard reaction coordinate for the analysis of biomolecular folding is the fraction of native structure formed, often called *Q* [7, 49, 50]. The SMOG-enhanced version of GROMACS v4.5 available on the SMOG website contains the tool “g_kuh,” which analyzes trajectories using native structural measures, including *Q*. Consistent with the experimentally-observed two-state folding dynamics of CI2 [51], plotting the free energy as a function of *Q* shows two basins at the folding temperature (T_F_). There is a folded basin at high *Q* and an unfolded basin at low *Q*, which are separated by a free-energy barrier (Fig 1C). Here, *Q* is defined as the fraction of natively-contacting residue pairs that are within 1.5 times their native distance.

### Using SMOG 2 to explore multiple levels of structural detail

**Fig 2:**
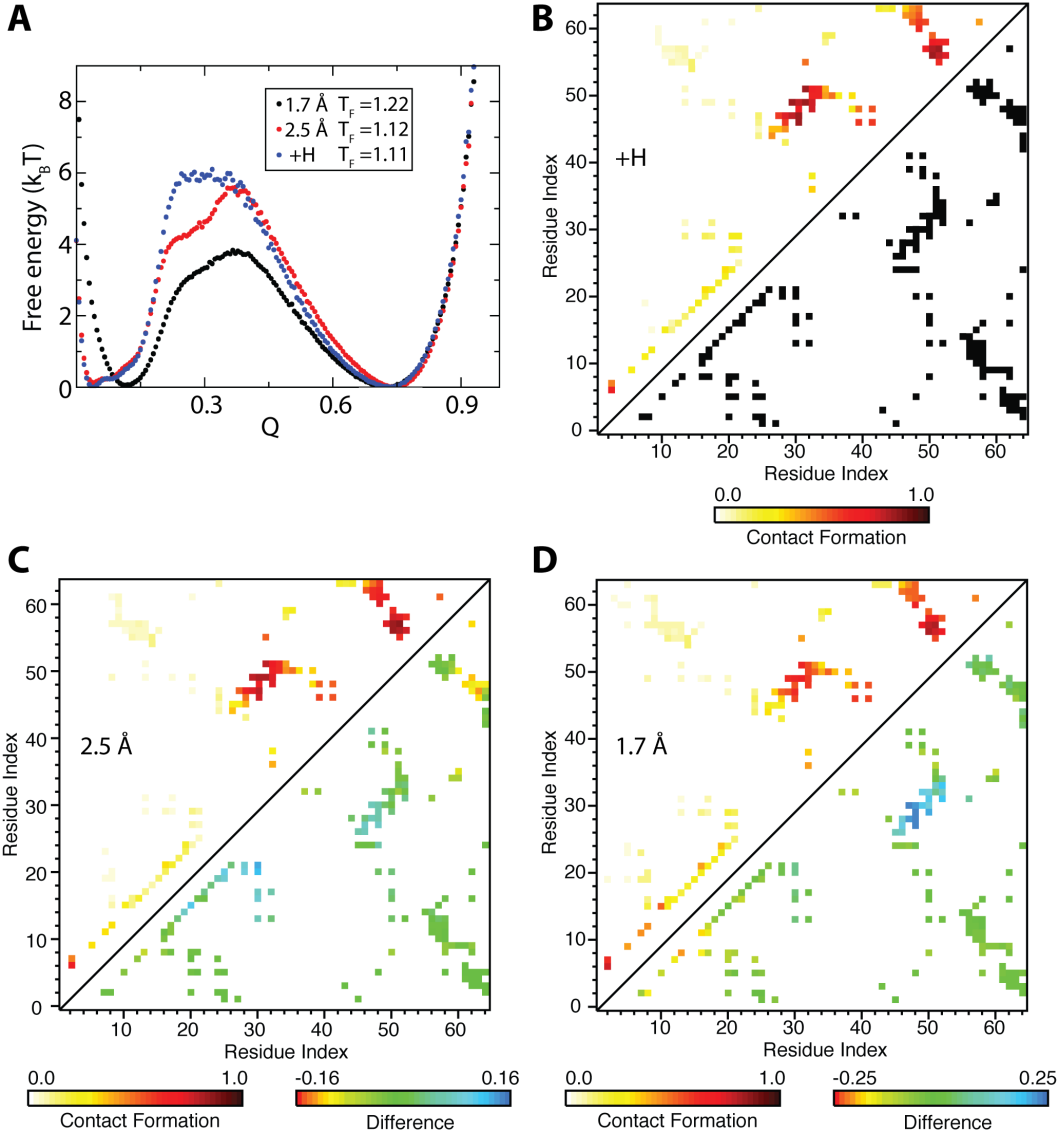
Composition of the folding TSE is robust to variations in the structural resolution of CI2. A) Free energy profiles as a function of the number of native atom-atom contacts *Q*, for three atomic geometries: uniform heavy atom diameter of 1.7 Å (M^1.7^ , black), uniform heavy atom diameter of 2.5 Å (M^2.5^, red), and heterogeneous heavy atom sizes with hydrogen excluded volume, +H (M^+H^, blue). The similar barrier height between M^2.5^ and M^+H^ suggests that the excluded volume in Amber99sb is roughly equivalent to an average of 2.5 Å diameter for heavy atom beads. Increasing the excluded volume raises the folding barrier and lowers T_F_ [41]. Note that the profile in Fig 1 is different because it was generated using the SBM_AA default of 2.1 Å diameter and a cutoff of 1.5 times the native distance to define a formed native contact, whereas a cutoff of 1.2 is used here. B) CI2 native contact map (lower triangle) and average contact formation at the unfolding side of the free-energy barrier at *Q* = 0.30 for M^+H^ (upper triangle). C) Comparison at *Q* = 0.30 of M^+H^ and M^2.5^. Average contact formation of M^2.5^ (upper triangle) and difference for each contact with positive values indicating higher formation in M^+H^ (lower triangle). D) Comparison at *Q* = 0.30 of M^+H^ and M^1.7^. Average contact formation of M^1.7^ (upper triangle) and difference for each contact with positive values indicating higher formation in M^+H^ (lower triangle).

In addition to models with C_*α*_ or all-atom resolution, SMOG 2 templates can be modified to describe any level of structural detail. For example, included in the distribution is a template that accommodates the explicit representation of hydrogens (Fig 2). This template, called “SBM_AA+hydrogen”, uses heterogeneous atomic radii modeled from the vdW parameters in the Amber99sb forcefield [38]. In contrast to the other included templates, there are multiple non-bonded types and associated changes, which can serve as an example of how to manipulate the SMOG 2 template syntax.

Here, we use the SBM_AA+hydrogen template to study protein folding with models that have identical native contact potentials, but differing levels of geometric detail. This provides a baseline test of the effects of atom size and molecular geometry on the folding landscape. The free-energy profiles along *Q* are shown for three SBMs of CI2, two with uniformly-sized heavy atoms of diameters 1.7 Å and 2.5 Å (parameter *σ*_NC_ in Eq 1), and one using the SBM_AA+hydrogen template (Fig 2A). These three models are denoted M^1.7^, M^2.5^, and M^+H^, respectively. Notably, increasing the excluded volume raises the folding barrier and lowers the folding temperature (T_F_) [41]. M^2.5^ has a folding barrier of the same height as M^+H^, though M^+H^ has a single flat barrier shape and M^2.5^ has a significant shoulder. The overall character of CI2 folding is consistent between the three models: two-state kinetics with a barrier centered around *Q* = 0.4. Differences in folding mechanism can be discerned by comparing the average contact formation in the barrier region. Visual inspection of average contact maps in the upper triangles of Fig 2 (panels B-D) shows that the transition state ensemble (TSE) is highly similar between the models. The detailed differences between M^+H^ and the models with uniform atom sizes are highlighted in the lower triangles of Fig 2 (panels C and D). M^+H^ versus M^2.5^ shows early formation of some secondary structure and delayed formation around ARG48 and ARG62. M^+H^ versus M^1.7^ mainly shows early formation of the parallel *β*-strand. Overall, while this analysis indicates that two-state folding and the character of the TSE are insensitive to the details of the atomic geometry in CI2, there are subtle effects on secondary structure formation and the shape of the free-energy barrier.

### Applications of SMOG 2 to large systems

To demonstrate of capacity of SMOG 2 to study systems of increased size, we used it to prepare simulations of the HIV-1 capsid shell. The HIV-1 capsid shell is composed of 1356 p24 proteins, which form hexameric and pentameric subunits. As noted in the original manuscript [52], the inherent plasticity of the p24 motif enables the formation of this heterogeneous assembly. Together, there are 216 hexameric units and 12 pentameric units that coincide with vertices of the assembly, which together form a “fullerene cone” shape. In total, there are 2.4 million non-hydrogen atoms in this system, making it the largest asymmetric structure available in the PDB. Previously, using explicit-solvent simulation of the full complex, it was found that the structural model maintained its structural integrity on the timescale of 100 ns [52]. This observation lends support to the details of the structural model, thereby implicating the formation of specific stabilizing interprotein interactions.

**Fig 3:**
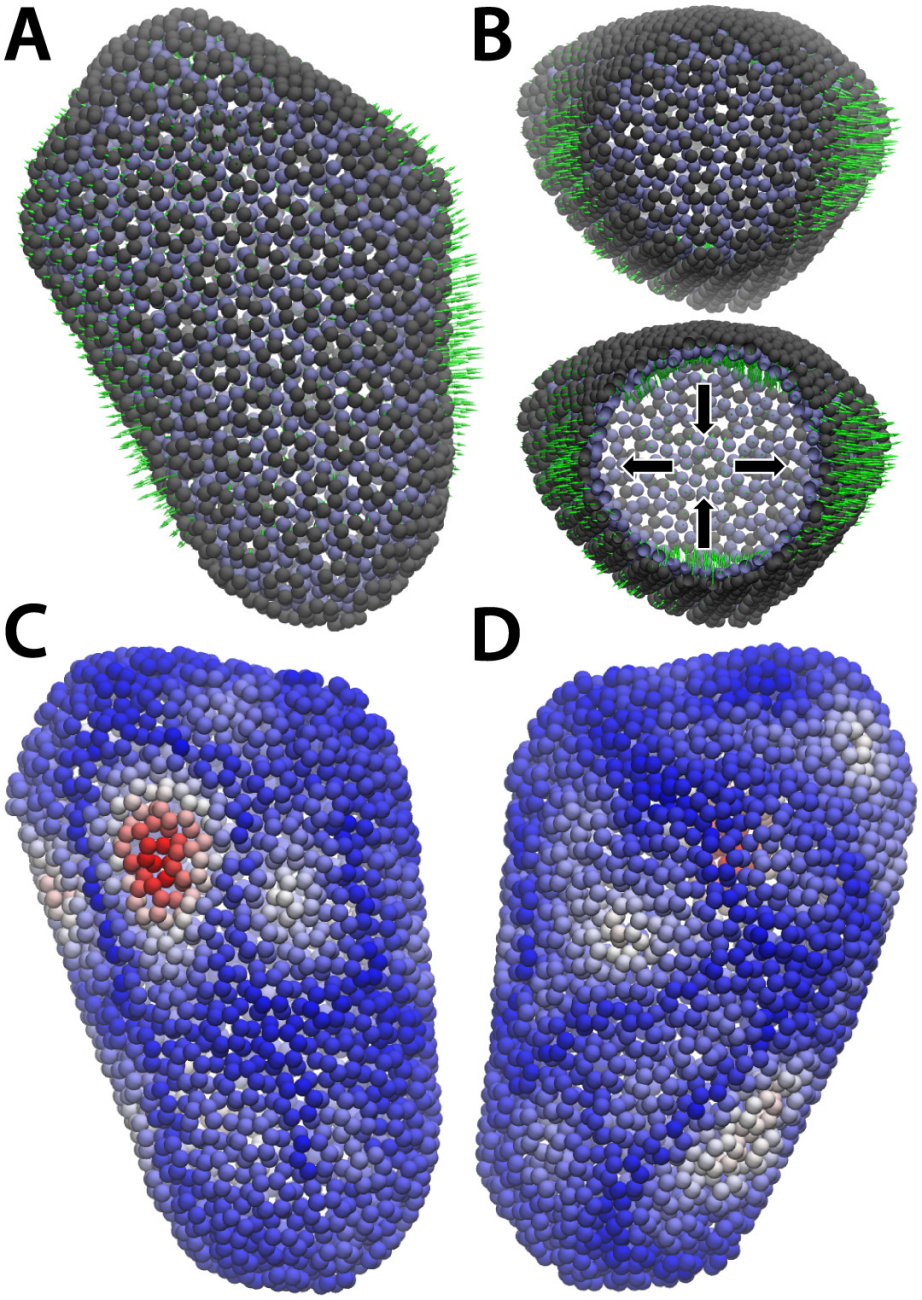
Correlated fluctuations are observed in all-atom simulations of the HIV-1 capsid. A) Side view of HIV-1 capsid with the center of mass of each domain shown as a grey (N-terminal) and ice blue (C-terminal) sphere. The first principal component is shown with green arrows (length of the arrows is not to scale). B) Same as panel (A), rotated 90°. The bottom panel shows the same complex with part of the system hidden. This reveals that, while a large number of domains move outwards (A), others move inward, resulting in a concerted breathing-like motion. C) Capsid shown with centers of mass colored by the scale of the motion in the second mode (blue: small, red: large). The largest fluctuations are centered around hexamer 1218. D) Rotated view of (C).

To elucidate the global motions of the mature HIV-1 capsid, we prepared a structure-based model with SMOG 2. Due to large number of chains and atoms, the web-based smog-server is not capable of processing this system. Since this system lacks global symmetry, it is important to simulate the full assembly in order to probe the dynamics. This is in contrast to more symmetric viral systems, where it may be possible to reduce the computational requirement by utilizing knowledge of the symmetry. From our simulation of the full complex, we performed principle component analysis (PCA) to identify the global modes of motion. Specifically, we calculated the center of mass of each domain of p24 (in total 2712 pseudoparticles) as a function of time and then evaluated the PCAs of the motions of the centers of mass. We find that the first two PCAs provide dominant contributions to the overall fluctuations of the complex, where the five largest eigenvalues were 7.7, 3.5, 2.9, 2.4, 1.7 nm^2^. Visualization of the first PCA (Fig 3) shows that the capsid exhibits an overall breathing-like motion. That is, there is correlated expansion and contraction of opposing sides of the capsid. With regards to the second mode, there was not a visible pattern in the direction of motion of the atoms. Nonetheless, when comparing the relative mobility of each domain we find that the largest fluctuations associated with this mode are centered around a specific hexamer (chain 1218 in the PDB file). Since subsequent conformational changes and disassembly are involved in HIV infection, this elevated degree of mobility suggests that this region may facilitate functional processes (e.g. recognition, or rupture propagation).

### Computational performance

For modestly sized systems (<20,000 atoms) the SMOG 2 program is lightweight and runs in under a minute on a desktop computer. The numbers quoted here use the template SBM_AA and are performed on a single core of a 2.30 GHz Intel Xeon E5-2630 CPU. For example, creating a topology file for adenylate kinase (1 chain, containing 1656 heavy atoms) takes 7 seconds and 124 MB of memory. While the largest systems considered in this manuscript take significantly more resources, topologies can easily be generated on modern desktop computers. SMOG 2 for the 70S ribosome (150 thousand atoms) takes 12 minutes and 3.1 GB of memory, and the HIV-I capsid (2.4 million atoms in 1356 chains, [52]) takes 89 minutes and 13.9 GB of memory.

**Fig 4:**
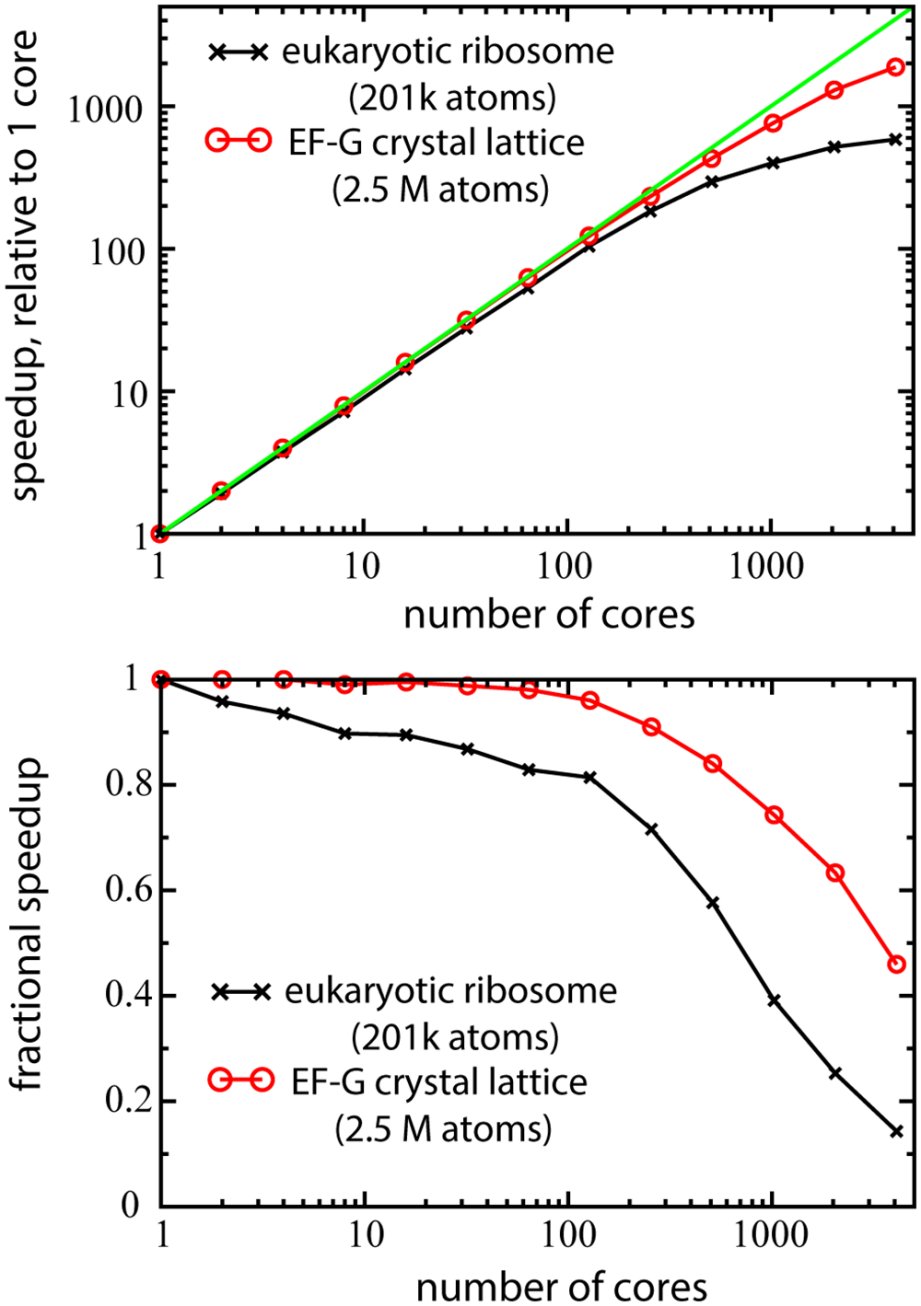
Large structure-based simulations are highly scalable. The two test systems are the eukaryotic ribosome containing 201 thousand atoms and a modeled 8x8x8 crystal lattice of EF-G containing 2.5 million atoms. Simulations used the SBM_AA template with GROMACS v4.6.3 and ran on the Stampede supercomputer located at the TACC.

Regarding the performance of MD simulations, SBMs exhibit strong scaling with parallelization on modern computing architectures. With GROMACS (v4 or v5), smaller simulations (<2000 atoms) can typically scale up to the number of processors available on a single motherboard, and larger simulations can significantly benefit from the combination of multiple compute nodes. The ribosome has previously been studied using SBMs [25], and it scales to ∼1000 cores on modern supercomputers (Fig 4). SBMs also exhibit weak scaling, which can be seen with the 2.5M atom EF-G lattice scaling to ∼2000 cores.

## Future Directions

There are many exciting applications for exploring the dynamics of biomolecules and molecular modeling that can be incorporated into the SMOG 2 infrastructure. Investigators are currently studying the entropic effects of post-translational modifications such as glycosylation [53] and the energetic effects of electrostatic interactions between nucleic acids and proteins [44, 54]. Another interesting development has been the integration of residue-level co-evolutionary information into structure-based potentials [55, 56]. Co-evolutionary information has a similar theoretical basis to SBMs in the “principle of minimal frustration” [1, 57], and they can help extend SBMs beyond the single-minimum paradigm [58]. With these new directions in mind, it is our intention that SMOG 2 will support the development of diverse applications of SBMs, by establishing a common framework that facilitates portability and collaboration.
